# Titanium dioxide nanotubes promote M2 polarization by inhibiting macrophage glycolysis and ultimately accelerate endothelialization

**DOI:** 10.1002/iid3.429

**Published:** 2021-04-09

**Authors:** Wen P. Yu, Jing L. Ding, Xin L. Liu, Guo D. Zhu, Feng Lin, Jian J. Xu, Ziyao Wang, Jian L. Zhou

**Affiliations:** ^1^ Department of Cardiovascular Surgery The Second Affiliated Hospital of Nanchang University Nanchang China; ^2^ Department of Gastroenterology The Second Affiliated Hospital of Nanchang University Nanchang China; ^3^ Department of Clinical Pathology The First Affiliated Hospital of Gannan Medical College Ganzhou China

**Keywords:** endothelialization, glycolysis, immune metabolism, macrophage polarization, titanium nanotube

## Abstract

Titanium has been widely used in prosthetic valves, but they are associated with serious defects in titanium‐based prosthetic valves, such as thrombosis, calcification, and decay. Therefore, it is very important to biofunctionalize titanium‐based valves to reduce inflammation and accelerate endothelialization of stents and antithrombosis. The titanium dioxide nanotubes were prepared from pure titanium (Ti) by anodic oxidation method in this study. The effects of titanium dioxide nanotubes on the metabolism of macrophages and the inflammatory reaction as implants were studied in vitro. The polarization state of macrophages and the ability to accelerate endothelialization were analyzed. The results demonstrated that titanium nanotubes promote M2 polarization of macrophages by inhibiting glycolysis and activating the Adenosine monophosphate‐activated protein kinase (AMPK) signaling pathway. In general, biofunctionalization titanium with nanotube could inhibit macrophage glycolysis, reduce inflammatory factor release and promote M2 polarization by activating the AMPK signaling pathway. And endothelialization was accelerated in vitro. Our result demonstrated that titanium nanotube could act as a potential approach to biofunctionlize titanium‐based prosthetic valves for endothelialization.

## Graphical abstract

Biofunctionalization titanium with nanotube could inhibit macrophage glycolysis, reduce inflammatory factor release and promote M2 polarization by activating the adenosine monophosphate‐activated protein kinase (AMPK) signaling pathway. And endothelialization was accelerated in vitro. Our result demonstrated that titanium nanotube could act as a potential approach to biofunctionlize titanium‐based prosthetic valves for endothelialization

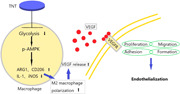

## 1 INTRODUCTION

Heart valve disease is a serious public health problem and its incidence is increasing day‐by‐day.^1^ The potential treatment for this involves the replacement of valves with biological, allogeneic, or mechanical prosthetic valves. They are widely used to replace the damaged heart valves and are considered the best treatment for patients with severe valve stenosis or regurgitation.^2^ However, the mechanical valve and biological valve replacements used in clinical practice have serious defects, wherein mechanical valve replacement needs lifelong anticoagulation and easily causes bleeding and embolism.

Titanium‐based biomaterials are widely used in vascular stents, thrombus filters, and other intravascular medical devices. However, implant failure often occurs due to surface thrombosis and endothelial dysfunction.^3^ In recent years, nanomaterials with unique nanotube array structures have attracted much attention in the fields of bone implantation, drug delivery, tumor treatment, and blood contact biomaterials.^4^ Nanotube arrays on titanium surface not only have good mechanical properties and corrosion resistance of titanium itself but also build unique nanotube structure on the surface of titanium to improve the biocompatibility. However, few studies on the effects of nanotube diameter and nanoarrays on endothelial cell behavior have been reported. Research has revealed that titanium nanotubes (TNT) with different diameters could promote M2 polarization of macrophages, release more vascular endothelial growth factor (VEGF), and accelerate endothelialization by activating ERK1/2 and PI3K/AKT signaling pathways.^5^ In addition, the relationship between TNT and immune cell metabolism is unclear.

Macrophages play an indispensable role in human immunity and they can produce different effector cells based on different surrounding environments.^6^, ^7^ The most representative phenotypes are M1 and M2. From the point of view of host defense, M1 macrophages have inflammatory properties and positive effects on microbial pathogens and tumor immunity, whereas M2 macrophages promote tissue repair and metabolic homeostasis, playing a key role in parasite immunity.^8^ However, the immune system constantly senses and responds to environmental threats that have a considerable bioenergy cost. Simultaneously, metabolic processes provide the basis for the synthesis of RNA, DNA, proteins, and membranes, which are necessary for the proliferation and activation of immune cells.^9^ In other words, the effects of cell metabolism on a series of physiological activities of immune cells are still unimaginable. This huge “energy cost” is mainly solved by glycolysis, tricarboxylic acid cycle, and oxidative phosphorylation. In the early 1990s, Bustos and Sobrino^10^ were the first to propose the inhibitory effect of glucocorticoids on the production of cytokines by macrophages, which might be due to the inhibition of glycolytic enzymes PKF1 and PKF2. M1 macrophages largely depend on aerobic glycolysis, whereas M2 macrophages mainly use fatty acid oxidation (FAO; β‐oxidation) to promote mitochondrial oxidative phosphorylation.^11^ These studies have fully demonstrated that the phenotype of macrophages can be transformed by regulating cell metabolism. Among them, glycolysis in M1 macrophages has a clear conclusion, but the relationship between glycolysis and M2 macrophages is not clear.

However, the relationship between TNT and glycolysis is not clear. In this study, TNT of different sizes (TNT20 and TNT60) were prepared at different voltages (20 and 60 V) to elucidate the mechanisms of intrinsic immune regulation, macrophage polarization, and accelerated endothelialization of TNT, as well as whether the diameter of the nanotubes is affected.

## 2 MATERIALS AND METHODS

### 2.1 Materials

Dulbecco's modified Eagle's medium (DMEM), Roswell Park Memorial Institute (RPMI 1640) medium, fetal bovine serum (FBS), 4′,6‐diamidino‐2‐phenylindole (DAPI) stain solution, and 0.25% trypsin‐EDTA solution was purchased from Sangon Biotech. Penicillin–streptomycin and Phorbol 12‐myristate 13‐acetate (PMA) were purchased from Solarbio Science & Technology Co., Ltd. Ammonium fluoride and ethylene glycol (Sinopharm chemical reagent). The cell counting kit‐8 (CCK‐8) solution and Matrigel (BD Biosciences). Lactic acid assay Kit (Colorimetric method) and Glucose assay Kit (O‐toluidine method) were purchased from Lengton Biotechnology. Anti‐Glut1, anti‐HK2, anti‐CD86, anti‐CD206, donkey anti‐rabbit secondary antibodies, anti‐AMPK‐α, and AMPK inhibitors were purchased from Abcam. A medical‐grade Ti rod was purchased from Baoti.

## 3 METHODS

### 3.1 Preparation and characterization of TNT

Circular piece with a diameter of 34 mm and thickness of 1 mm was prepared by Ti. The anodic oxidation method was used to prepare TNT.^12^ Briefly, Ti was ultrasonically treated with ethanol, acetone, and double‐distilled water, followed by polishing for 1 min with polishing water containing HF, HNO_3_, and H_2_O (volume ratio: 1:4:5). At different voltages (20 and 60 V), Ti was placed in an electrolyte solution consisting of ammonium fluoride (2.6 g), double‐distilled water (50 ml), and ethylene glycol (450 ml for 60 min. The titanium substrate that has not been electrolyzed was used as a control (marked as Ti). Before sowing the cells, the plate was exposed to ultraviolet radiation for 1 h. The surface morphology and topography of Ti samples were characterized by a scanning electron microscope (SEM; JSM‐6700F; JEOL).

### 3.2 Cell culture

THP1 (Human acute monocytic leukemia cells) and HUVECs (human umbilical vein endothelial cells) were cultured. THP1 cells were purchased from the cell bank of the Chinese Academy of Sciences, and HUVECs were purchased from ATCC. THP1 cells were cultured in RPMI medium containing 10% FBS under standard conditions (5% CO_2_, 37°C, and 100% humidity). Before use, THP1 cells were treated with PMA (100 ng/ml). The cells were then inoculated on Ti, TNT20, and TNT60. After 3 days of culture, the medium was collected and mixed with high‐glycemic DMEM at a ratio of 1:1 to prepare conditioned medium (CM). HUVECs were cultured in DMEM medium containing 10% FBS under standard conditions. After the cells reached a confluence of 85%, 0.25% trypsin‐EDTA was used for subculturing.

### 3.3 Effects of TNTs on glycolysis of macrophages

#### 3.3.1 Glucose and lactate assays

The cells were placed in 12‐well plates with different stimulus materials in different plates (blank control, Ti, TNT20, and TNT60), and the supernatant was obtained after incubation for 24 h. Both assays were performed according to the instructions of the kits. The absorbance of the color showed linear relation to the content of lactic acid at 530 nm. The principle of the glucose detection kit is that glucose is condensed with O‐toluidine in hot acetic acid solution to form a blue schiff base with an absorption peak at 630 nm. For measurements, high glucose DMEM medium was used. Glucose consumption was calculated by subtracting the amount of glucose in the sample from that in the medium without cells. Lactate production was calculated by subtracting the concentration of any lactate in the medium without cells from that of the samples. Glucose and lactate assays were performed in parallel.

#### 3.3.2 Quantitative polymerase chain reaction (PCR) analysis

Total RNA was isolated with Trizol reagent (Thermo Fisher Scientific) and reverse‐transcribed with EASTWIN system according to the manufacturer's instructions. The expression levels of GLUT1 and HK2 were detected by real‐time quantitative PCR (RT‐qPCR) using StepOne real‐time PCR system (Applied Biosystems) and β‐actin was used as an internal reference gene. The primers used for amplification in this study are summarized in Table 1 (Generay Biotech).

**Table 1:** Primer used in this study

Gene and primer direction		Sequence (5′–3′)
Human β‐actin	Forward	GACGGCCAGGTCATCACTATTG
	Reverse	AGGAAGGCTGGAAAAGAGCC
Human ARG	Forward	GTGGAAACTTGCATGGACAAC
	Reverse	AATCCTGGCACATCGGGAATC
Human CD206	Forward	TCCGGGTGCTGTTCTCCTA
	Reverse	CCAGTCTGTTTTTGATGGCACT
Human iNOS	Forward	TTCAGTATCACAACCTCAGCAAG
	Reverse	TGGACCTGCAAGTTAAAATCCC
Human IL‐1β	Forward	ATGATGGCTTATTACAGTGGCAA
	Reverse	GTCGGAGATTCGTAGCTGGA
Human VEGF	Forward	ATCGAGTACATCTTCAAGCCAT
	Reverse	GTGAGGTTTGATCCGCATAATC
Human Glut1	Forward	CACCGCTATGGGGAGAG
	Reverse	CCACAGAGAAGGAGCCAA
Human HK2	Forward	CTGAGGACATCATGCGAGGCA
	Reverse	GTGGCAGGGGAACGAGAAGGT

Abbreviations: ARG, arginase; CD206, mannose receptor; Glut1, Glucose transporter type 1; HK2, hexokinase 2; IL, interleukin; iNOS, inducible nitric oxide synthase; VEGF, vascular endothelial growth factor.

#### 3.3.3 Western blot assay

Proteins were extracted with M‐PER mammalian protein extraction reagent (Thermo Fisher Scientific), and their concentration was measured by the BCA Protein Assay Kit (Thermo Fisher Scientific). Western blotting was used to detect the protein expressions of GLUT1 and HK2. Fifteen microliters of each cell extract were separated by electrophoresis and transferred onto the polyvinylidene fluoride membrane. Second, the membrane was blocked with 5% bovine serum albumin for 1 h and incubated with primary antibodies overnight. The membrane was then washed three times and incubated with donkey anti‐rabbit secondary antibodies (1:100) at room temperature for 1 h. Finally, the membrane was exposed using a TANON imager.

#### 3.3.4 Macrophage polarization

Macrophage polarization was observed by indirect immunofluorescence staining before undergoing a confocal laser scanning microscope (CLSM). Rabbit anti‐human CD86 (1:100) was used as a proinflammatory marker, and rabbit anti‐human CD206 (1:50) was used as an anti‐inflammatory marker. The cells were immobilized in paraformaldehyde at 4°C for 10 min, and then treated with 0.1% Triton X‐100 (Sigma‐Aldrich) for 10 min. After blocking with 5% bovine serum albumin, the cells were incubated with primary antibodies overnight at 4°C. Next, the donkey anti‐rabbit immunoglobulin G secondary antibodies (1:100) were used to visualize the cells. The nuclei were stained with DAPI (1 mg/ml), In short, after the above steps, the liquid was sucked out, washed twice with phosphate‐buffered saline (PBS), and DAPI staining solution was added. The liquid was dyed at room temperature for 15 min in the dark. all samples were observed by CLSM.

#### 3.3.5 Inflammatory gene expression

Two M1‐related genes, interleukin‐1 (IL‐1) and iNOS, and two M2‐related genes, ARG1 and CD206 were detected by RT‐qPCR. In addition, the messenger RNA (mRNA) levels of VEGF were detected. The relative gene expression was normalized with the housekeeping gene β‐actin. The PCR primers used are listed in Table 1.

#### 3.3.6 Inflammatory cytokines detection

Subsequently, proinflammatory cytokines (IL‐1β and IL‐8) and anti‐inflammatory cytokines (ARG and IL‐10) were examined with chemiluminescence. Macrophage supernatants were collected and then the supernatant was detected with the corresponding detection kit (Sangon Biotech), according to the manufacturer's instructions (which included the IL‐1 kit, the IL‐8 kit, the ARG kit, the IL‐10 kit, and the VEGF kit). Finally, IMMULITE 1000 was used for analysis.

### 3.4 Effects of macrophage conditioned medium on endothelialization of HUVECs

#### 3.4.1 HUVECs adhesion evaluation

The conditioned medium for macrophages treated with three materials (Ti, TNT20, and TNT60) was collected for use. HUVECs were cultured in 96‐well plates at a density of 2 × 10^5^ cells/ml in CM. After 2, 6, or 12 h, the wells were rinsed three times with PBS. The CCK‐8 solution was used to culture the adherent cells for 2 h and read at a 450‐nm filter.

#### 3.4.2 Evaluation of HUVECs proliferation

To assess the proliferation of HUVECs in different conditioned medium, 2 × 10^4^ cells were seeded in 96‐well plates. After 1, 3, or 5 days, 10 µl CCK‐8 solution was added to each well. The cells were incubated for 2 h, and then the absorbance was measured at 450 nm with a microplate reader.

#### 3.4.3 Scratch assay

Scratch assay^13^ was used to assess the migratory ability of endothelial cells. HUVECs were seeded in a six‐well plate. When the cells reached 100% confluence, a monolayer of the cells was scratched with a pipette, and then washed three times with PBS. The conditioned medium was added and the results at 0 and 6 h were observed. ImageJ software was used to assess the area of the scratch.

#### 3.4.4 Tube formation assay

After HUVECs were cocultured with Matrigel (Matrigel is a soluble basement membrane extract extracted from Engelbreth‐Holm‐Swarm mouse sarcomas that can be polymerized into a bioactive matrix material), the capillary‐like structures were formed.^5^ The 96‐well plates were coated with 50 µl matrigel and then incubated at 37°C for 30 min. HUVECs were seeded at a density of 4 × 10^5^/ml with 100 µl conditioned medium and imaged after incubation for 6 h. ImageJ software was used to evaluate the branch points and total tube length of each field.

### 3.5 Mechanism of TNT‐inhibiting glycolysis

To explore the mechanism of TNT affecting glycolysis of macrophages, the phosphorylation of classical adenosine monophosphate‐activated protein kinase (AMPK) signaling pathway related to metabolism was detected. To verify whether the AMPK signaling pathway was passed, AMPK inhibitors were added for comparison. The expression of related proteins was detected by Western blot analysis. The conditioned media were collected for each group to evaluate the effects of HUVECs on a range of physiological activities (proliferation, adhesion, migration, and formation). At the same time, the amount of VEGF released by the macrophages from the supernatant collected from each group was detected. The mRNA expression of VEGF was quantitatively analyzed by RT‐PCR.

#### 3.5.1 Statistical analysis

The data were analyzed using GraphPad Prism8 and presented as means ± *SD*. One‐way analysis of variance was used to determine significant differences. The probability value of less than .05 (*p* < .05) was considered to be statistically significant.

## 4 RESULT

### 4.1 Characterization of TNT surfaces

**Figure 1:**
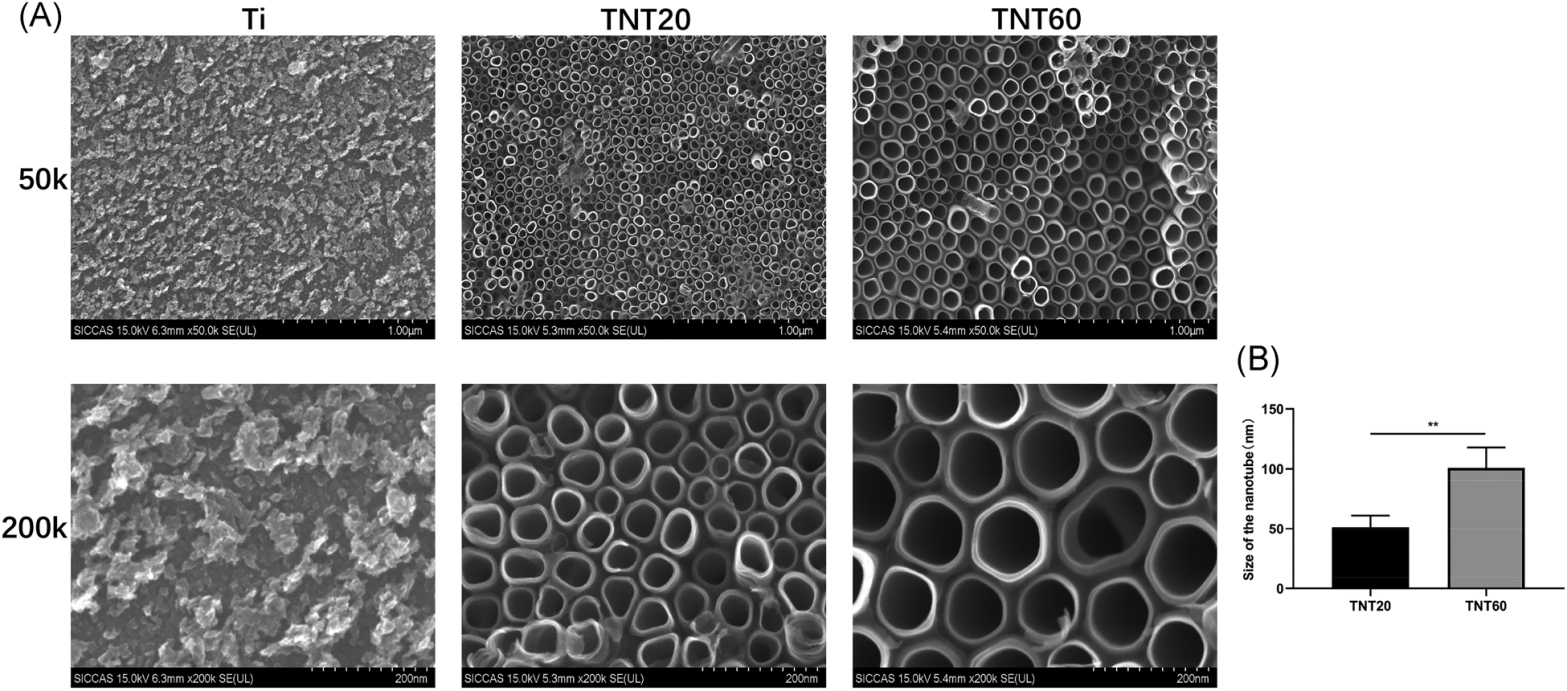
Characterization of titanium dioxide nanotubes. (A) The surface morphologies of three samples. (Ti, TNT20, and TNT60) observed using scanning electron microscopy at different magnifications. (B) The diameters of the nanotubes. Results are presented as mean ± *SD* (*N* = 3). Ti, pure titanium; TNT, titanium nanotubes; *p < .05 and **p < .01

Titanium was treated by anodic oxidation at different voltages (20 and 60 V), and then the surface morphology was observed under SEM. As shown in Figure 1A, regular nanoarrays were observed on the surfaces of TNT20 and TNT60 when compared to those with pure titanium plates (Ti), and the diameter of the nanotubes was increased with increasing voltage. After ImageJ software analysis, the diameter of the nanotubes were 51.453 ± 9.629 nm (TNT20) and 100.969 ± 16.877 nm (TNT60) (Figure 1B).

### 4.2 Effects of TNTs on glycolysis of macrophages

**Figure 2:**
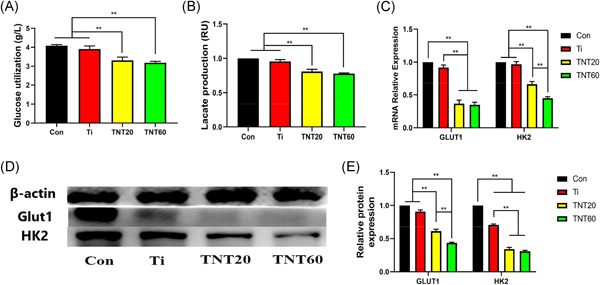
Effects of TNTs on glycolysis of macrophages. Culture media of each group (Control, Ti, TNT20, and TNT60) stimulated macrophages were collected for the measurement of glucose (A) and lactate (B). (C) Gene expressions of Glut1 and HK2 in macrophages stimulated by each group (Control, Ti, TNT20, and TNT60) for 48 h were measured by quantitative real‐time polymerase chain reaction. (D) Protein expressions of Glut1 and HK2 in macrophages stimulated by each group (Control, Ti, TNT20, and TNT60) for 48 h were measured by Western blot analysis. (E) Analysis of HK2 and Glut1 protein expressions using the images shown in (D). Results are presented as mean ± *SD* (*N* = 3). Ti, pure titanium; TNT, titanium nanotubes. *p < .05 and **p < .01

The relationship between TNTs and macrophage polarization has been described in many studies, both in modified TNTs. Previous studies have reported that nano‐arrays on the surface of titanium plates can regulate macrophage polarization towards M2‐type by activating autophagy.^14^ Wang et al.^15^ have also found that TNTs of different diameters could regulate the polarization of macrophages. The results revealed that the TNTs regulated macrophage polarization by influencing cell metabolism. Therefore, glucose utilization and lactate production were measured as shown in Figure 2A,B. Compared with CON and Ti, the glucose utilization of TNT20 and TNT60 was reduced, which were 3.306 ± 0.174 and 3.184 ± 0.078 g/L, respectively (Figure 2A), and similarly, the lactic acid production, which were 0.810 ± 0.033 (TNT20) and 0.780 ± 0.008 (TNT60) (Figure 2B), and these indicators reflect the level of glycolysis. To further verify whether TNT inhibits glycolysis of macrophages, two genes that represent glycolysis levels and measurement of mRNA and protein expression levels were selected, respectively. The results showed that expression of both mRNA and protein was inhibited (Figure 2C,D), and it was believed that TNT somehow inhibits macrophage glycolysis.

### 4.3 Macrophage polarization analysis

**Figure 3:**
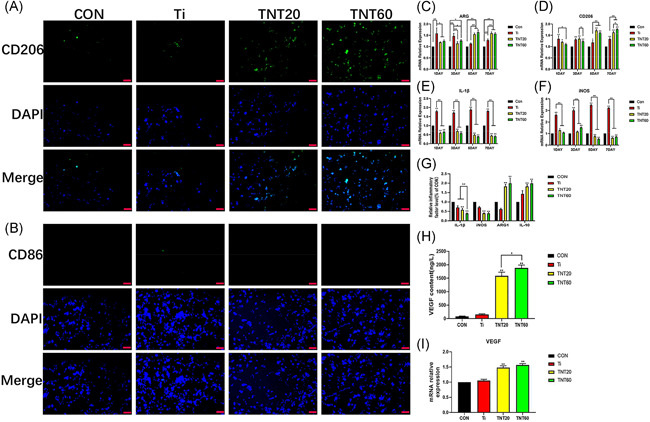
Macrophage behavior on titanium dioxide nanotubes. (A) Confocal detection of the M2 marker protein CD206 after 48 h of culture. (B) Confocaldetection of the M1 marker protein CD86 after 48 h of culture. (C–F) Real‐time polymerase chain reaction detection of macrophage polarization and inflammation‐related gene expression after 24 h of culture. (G) Detection of inflammatory factors in supernatants. (H) ELISA of macrophage‐secreted VEGF in supernatants after 72 h of culture. (I) VEGF gene expression levels in macrophages after 24 h of culture. Results are presented as mean ± *SD* (*N* = 3). ARG, arginase; CON, control; DAPI, 4′,6‐diamidino‐2‐phenylindole; ELISA, enzyme‐linked immunosorbent assay; IL, interleukin; iNOS, inducible nitric oxide synthase; mRNA, messenger RNA; Ti, pure titanium; TNT, titanium nanotubes; VEGF, vascular endothelial growth factor. *p < .05 and **p < .01

After detecting many indicators from the above, TNT was known to inhibit the glycolysis of macrophages. To determine whether the inhibition of TNT on glycolysis has any effect on macrophage polarization, the expression of M1‐and M2‐related genes was detected, various inflammatory mediators in the supernatant of each group of materials were quantitatively analyzed, and M1 and M2 macrophages were located by immunofluorescence. RT‐PCR results showed that the expression of M1‐related genes IL‐1β and iNOS was decreased mostly in the late stage (5–7 days) (Figure 3E,F). As shown in Figure 3C,D, M2‐related genes ARG and CD206 were upregulated at all time points, but the highest upregulation occurred on Days 5–7. The results of RT‐PCR showed that macrophages had a tendency of M2 polarization. To further verify this conclusion, the supernatant from each group was collected to detect inflammatory factors (IL‐1β, IL‐8, ARG1, and IL‐10). IL‐1β and iNOS, which are mainly secreted by M1 macrophages, were significantly lowered in TNT20 and TNT60 groups. In contrast, ARG1 and IL‐10 secreted by M2 macrophages were increased (Figure 3G). Microscopically, the Ti group demonstrated a stronger fluorescence when the M1‐related anti‐CD86 antibody was incubated with the sample (Figure 3B). However, when the samples were incubated with CD206 antibody, TNT20 and TNT60 showed stronger fluorescence, and TNT60 was more significant than TNT20 (Figure 3A). Moreover, the results of enzyme‐linked immunosorbent assay (ELISA) and RT‐PCR revealed that the supernatant of TNT20 and TNT60 contained more VEGF, and the gene expression level of VEGF was higher than that of other groups (Figure 3H,I). These findings suggest that TNT changes the polarization (towards M2) of macrophages by affecting the glycolysis level of macrophages.

### 4.4 Evaluation of the behaviors of HUVECs stimulated with conditioned medium (CM)

**Figure 4:**
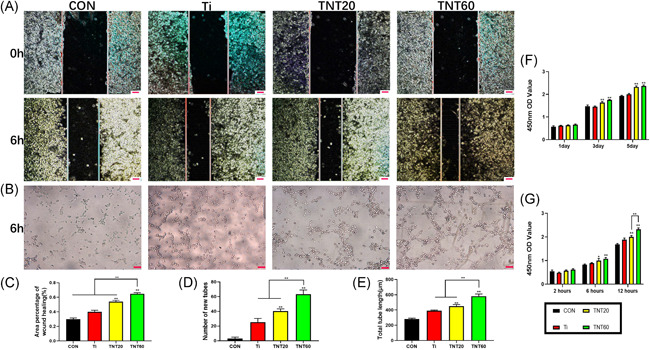
Evaluation of the in vitro behaviors of HUVECs stimulated with conditioned medium. (A) Representative images of HUVEC migration. (B) Representative images of tube formation for 6 h. (C) The percentage of coverage after 6 h of wound healing. (D) Quantitative analysis of the number of new tubes. (E) Quantitative analysis of the lengths of new tubes. (F) Cell proliferation in HUVECs for 1, 3, and 5 days. (G) Cell adhesion in HUVECs after 2, 6, and 12 h. Results are presented as mean ± *SD* (*N* = 3). CON, control; HUVEC, human umbilical vein endothelial cell; Ti, pure titanium; TNT, titanium nanotubes. *p < .05 and **p < .01

Cell migration, tube formation, proliferation, and adhesion were measured to determine the rate of endothelialization induced by TNT surface nanoarray via macrophages. As shown in Figure 4A, CM from TNT20 and TNT60 enhanced the migration of HUVECs when compared to the other two groups. The migration rate of each group was 29.8% ± 1.7% (CON), 39.8% ± 2.5% (Ti), 53.9% ± 1.8% (TNT20), and 64.8% ± 1.5% (TNT60) (Figure 4C). At the same time, the capillary‐like structure was found in TNT20 and TNT60 groups, and more significantly found in the TNT60 group (Figure 4B). In addition, the total length and the number of new tubes in each group were quantitatively analyzed, which were CON < Ti < TNT20 < TNT60 (Figure 4D,E). The proliferation and adhesion of endothelial cells were assessed by CCK‐8 solution. As shown in Figure 4F,G, endothelial cells showed strong proliferation ability on Day 3, especially in TNT20 and TNT60 groups. Similarly, endothelial cells in each group showed stronger adhesion at 12 h, and the TNT60 group was more significant than the other groups. These results indicate that conditioned media from TNT20 and TNT60 accelerate endothelialization in vitro, especially, the TNT60 group.

### 4.5 Effects of AMPK pathway inhibitors on endothelialization

**Figure 5:**
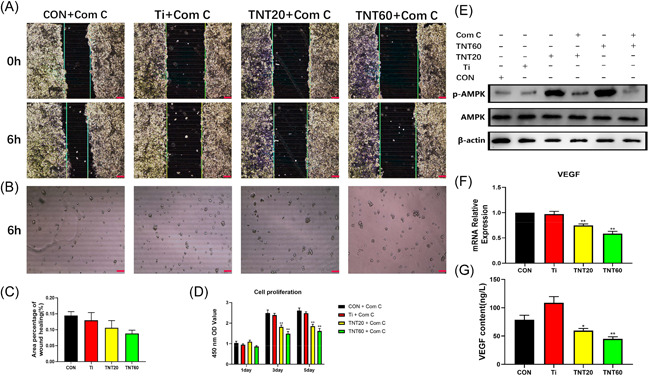
Effects of AMPK pathway inhibitors on endothelialization. (A) Representative images of HUVEC migration. (B) Representative images of tube formation for 6 h. (C) The percentage of coverage after 6 h of wound healing. (D) Cell proliferation in HUVECs for 1, 3, and 5 days. (E) Determination of AMPK phosphorylation in macrophage by Western blot analysis. (F) VEGF gene expression levels in macrophages after 24 h of culture. (G) ELISA of macrophage‐secreted VEGF in supernatants after 72 h of culture. Results are presented as mean ± *SD* (*N* = 3). AMPK, adenosine monophosphate‐activated protein kinase; COM ‐C: compound C, an AMPK signaling pathway inhibitor; CON, control; ELISA, enzyme‐linked immunosorbent assay; HUVEC, human umbilical vein endothelial cell; mRNA, messenger RNA; Ti, pure titanium; TNT, titanium nanotubes; VEGF, vascular endothelial growth factor. *p < .05 and **p < .01

**Figure 6:**
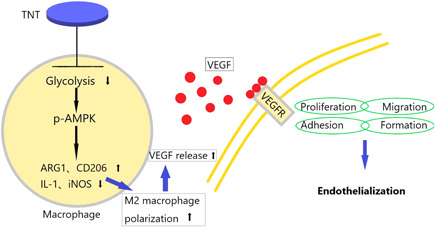
The schematic diagram of this study. AMPK, adenosine monophosphate‐activated protein kinase; ARG, arginase; IL, interleukin; iNOS, inducible nitric oxide synthase; TNT, titanium nanotubes; VEGF, vascular endothelial growth factor; VEGF, vascular endothelial growth factor receptor

After each group of materials (Ti, TNT20, and TNT60) was cultured with macrophages, AMPK inhibitors were added. CM were collected for each group to assess the effects of AMPK inhibitors on endothelialization. From Figure 5A–C, CM with inhibitor inhibited the migration and tube formation of endothelial cells. Moreover, the proliferation of endothelial cells was shown to be greatly hindered (Figure 5D). So, the phosphorylation of AMPK was evaluated by Western blot analysis. As shown in Figure 5E, both TNT20 and TNT60 activated AMPK phosphorylation, and when AMPK inhibitors are added, their phosphorylation levels are significantly reduced. According to the results of ELISA and RT‐PCR, VEGF content in the supernatant and gene expression level of TNT20 and TNT60 were lower than those of the other two groups (Figure 5F,G). The schematic diagram of this study was depicted in Figure 6. These data suggest that TNT (TNT60 is more significant than TNT20) inhibits glycolysis of macrophages, activates AMPK signaling pathway, and then promotes M2 polarization of macrophages, finally accelerating endothelialization.

## 5 DISCUSSION

Many studies have discussed the importance of Ti implants in anti‐inflammatory response and emphasized their role in surface nanoarrays.^1^, ^15^, ^16^, ^17^ The importance of hydrophilicity for implants is beyond doubt. TNT60 exhibited good hydrophilicity.^5^ Acid treatment increased the hydrophilicity of TNTs,^18^ and the larger the diameter of TNT, the better is the hydrophilicity.^19^ This is mainly due to the electrostatic interaction between positively charged material surfaces and negatively charged cells, which remains conducive to cell adhesion.^20^ It is important to note that TNT can upgrade nitric oxide and VEGF secretion at a suitable dimension, whereas the latter is the key step involved in endothelial cell function.^21^ Recent studies have shown that rapid endothelialization can reduce platelet adhesion.^22^

Metabolism promotes development, proliferation, differentiation, and effects or functions performed by the cells and tissues in adult organisms. The glycolytic metabolic pathway begins with the uptake of extracellular glucose from the cellular environment, followed by intracellular processing of glucose in the cytoplasm to produce pyruvate and many other products. Glucose utilization mainly relies on a series of multiple key enzyme reactions, eventually leading to the production of lactic acid and two net adenosine triphosphate (ATP) molecules as a source of energy. Several studies have pointed out glycolysis as being crucial for immune cell function.^23^, ^24^ This might come as a surprise, as glycolysis is not the most common way for the production of ATP. It is also seen that glycolysis can induce rapid activation of enzymes involved in this pathway. In contrast, oxidative phosphorylation, which produces more ATP, is a slower and more complex process, which results in physiological activity that requires rapid ATP production shifts to glycolysis.^25^ Moreover, GLUT1 acts as a speed‐limiting glucose transporter in proinflammatory polarized macrophages. Studies have shown that overexpression of GLUT1 in mouse macrophage cell line Raw264.7 leads to significantly increased glucose uptake and metabolism.^26^ In this study, TNTs were cocultured with macrophages to detect the above experimental indicators. TNT was shown to have an inhibitory effect on the glycolysis of macrophages.

A potential link between glycolysis and M2 macrophage activation is by the production of pyruvate from glycolysis, which then feeds into the tricarboxylic acid cycle to facilitate Ac‐CoA synthesis and histone acetylation^27^ or mitochondrial OXPHOS.^28^ Studies have shown that there are macrophages and multinucleated cells in the scaffold interface of Ti implants during the early stage of implantation.^29^, ^30^ Inappropriate macrophage activation can exacerbate inflammation and hinder the integration of healthy tissue/implant, and proper guidance of macrophage activation contributes greatly to implant performance and tissue healing.^30^, ^31^ Excessive release of IL‐8 also destroys the growth of endothelial cells,^32^ causing a decrease in endothelial cells and formation of a platelet thrombus. In contrast to IL‐8, increased IL‐10 secretion can effectively inhibit the production of proinflammatory cytokines.^33^ Tossetta et al.^34^ have found that IL‐1 and transforming growth factors can disrupt the tight connections of endothelial cells. In addition, with increased ARG1 and IL‐10 levels, the TNT surface nanoarray likely induces healing‐related M2 polarization.^35^ It is worth mentioning that VEGF acts as a key regulator of endothelialization and can synergistically promote tissue regeneration. VEGF is the most effective factor that promotes endothelialization to date.^36^ TNT60 produced more VEGF in our study, and therefore, the inflammatory response produced by TNT surface macrophages in this study is conducive for integrating implants and tissues.

The vascular lumens of endothelial cells regulate the dynamic pathways of nutrients and cells. In adults, the process of neovascularization begins with the release of graded VEGF in a nutrient‐poor, ischemic/anoxic environment, rebuilding the blood flow in this environment.^37^ In an animal study of vascular injury, shedding of endothelial cells leads to thrombus formation of platelets.^38^ Endothelial cell growth, including proliferation, adhesion, migration, formation, and secretion of products on valves, plays a vital role in the prevention of blood clotting.^39^

AMPK inhibits the synthesis pathway and activates the catabolic pathway to maintain energy homeostasis.^40^ M2 macrophages have the ability to highly activate AMPK,^41^ which is a key sensor of cell energy state, as well as a key factor for phosphorylation and FAO induction.^42^ AMPK activation can skew the macrophages towards the M2 phenotype, which is manifested as an increase in CD206 and a decrease in iNOS expression.^43^ This is consistent with our study results. Palsson‐McDermott et al.^44^ have proved that activation of PKM2 can reduce the inflammatory response and promote M2 macrophage polarization. However, AMPK is the only effective pathway found and other signaling pathways that are involved in regulation need further study.

It is well recognized that M1‐polarized macrophages increase glycolysis to rapidly trigger the microbicidal activity. However, it is unclear as to how the glycolytic activity is regulated in M2 macrophage polarization. There are few studies on the relationship between TNTs and macrophage metabolism. Our data indicated that TNT inhibits glycolysis of macrophages and activates AMPK signaling pathway, promoting macrophage polarization. It is possible to manipulate macrophage metabolism to promote immune responses, or indeed suppress inflammation in cases with autoimmunity. If this reprogramming should be sustained, perhaps through the epigenetic changes downstream of metabolic changes, then it might promote remission of chronic inflammatory diseases. Till now, many studies have focused on the drug loading capacity of TNTs only, and the purpose of regulating macrophage polarization involves the release of drugs.^45^, ^46^ However, the characteristics of TNTs are largely ignored. Hence, TNTs with different diameters were prepared, and the ability of these in regulating macrophage polarization was done by studying its effect on the glycolysis of macrophages, analyzing the polarization state of macrophages, and simulating the acceleration of endothelialization in vitro. Our data showed that the anti‐inflammatory and endothelial ability of TNT is directly related to its diameter. Large size TNT60 significantly reduces the release of proinflammatory factors and provides an anti‐inflammatory microenvironment that is conducive to tissue healing. Studies have shown that TNTs with a diameter of 120 nm can effectively inhibit the growth of proinflammatory cells and the release of proinflammatory factors.^47^ These results were in line with our study findings. It is likely that the surface nanoarrays affect the hydrophilicity of the material, and this requires further study. Metabolism is so important to immune cells, and it is necessary to find if TNT affects metabolism. Of course, our study is superficial at present and should explore the mechanism of TNT's influence on cell metabolism more deeply. For effective valve replacement, a better microenvironment for the implant to be integrated with the tissue is required. TNTs loaded with the drug might be effective, but they do not last long. It would be an exciting achievement if the intrinsic immune regulation of titanium dioxide nanotubes could enable implant and tissue integration. However, this is only a preliminary study, and further in vivo experiments are warranted to provide more evidence for this conclusion.

## 6 CONCLUSION

Our results suggest that TNT has intrinsic immunomodulatory effects, and it inhibits the glycolysis of macrophages by activating the AMPK signaling pathway. This in turn causes macrophages to produce fewer inflammatory factors and promotes M2 polarization. In addition, more VEGF is secreted to accelerate endothelialization. The larger the size of TNT (TNT60), the stronger the effects are. Our result demonstrated that titanium nanotube could act as a potential approach to biofunctionlize titanium‐based prosthetic valves for endothelialization.

## CONFLICT OF INTERESTS

The authors declare that there are no conflict of interests.

## AUTHOR CONTRIBUTIONS

Wen P. Yu carried out the experiments. Xin L. Liu prepared the manuscript. Jian L. Zhou and Jing L. Ding designed the experiments. Jian J. Xu and Ziyao Wang analyzed the experimental results. Feng Lin and Guo D. Zhu revised the manuscript. All authors reviewed the manuscript. All authors contributed to data analysis, drafting or revising the article, gave final approval of the version to be published, and agree to be accountable for all aspects of the work.

## Data Availability

All data are freely available.
